# Feasibility of an Internet-Based Intervention to Promote Exercise for People With Spinal Cord Injury: Observational Pilot Study

**DOI:** 10.2196/24276

**Published:** 2021-06-09

**Authors:** Christa Ochoa, Maria Cole, Katherine Froehlich-Grobe

**Affiliations:** 1 Baylor Scott & White Institute for Rehabilitation Baylor Scott & White Research Institute Baylor Scott & White Health Dallas, TX United States; 2 Craig Hospital Englewood, CO United States

**Keywords:** spinal cord injury, lifestyle intervention, physical activity, health promotion, eHealth

## Abstract

**Background:**

People with spinal cord injury (SCI) are less likely to be physically active and have higher chronic disease risk than those in the general population due to physical and metabolic changes that occur postinjury. Few studies have investigated approaches to promote increased physical activity (PA) for people with SCI despite evidence that they face unique barriers, including lack of accessible transportation and exercise equipment. To address these obstacles, we adapted an evidence-based phone-delivered intervention that promoted increased PA among people with SCI into a web-based platform, titled the Workout on Wheels internet intervention (WOW*ii*). The adapted program provides participants with weekly skill-building information and activities, basic exercise equipment, and ongoing support through weekly group videoconferencing.

**Objective:**

This pilot study was conducted to assess the feasibility of using a web-based and virtual format to deliver the WOW*ii* program in a randomized controlled trial.

**Methods:**

We assessed the feasibility of the web-based program by delivering an abbreviated, 4-week version to 10 participants with SCI. Rates of weekly videoconference attendance, activity completion, and exercise activity as tracked by an arm-based activity monitor were recorded for all participants.

**Results:**

Participants averaged 3.3 of 4 (83%) weekly group videoconferences attended, 3.4 of 4 (85%) web-based module activities completed, and 2.3 of 4 (58%) weeks of using the arm-based activity monitor. The majority of the sample (9/10, 90%) synced their arm-based PA monitor at least once, and overall engagement as an average of each component across the 4 weeks was 75%.

**Conclusions:**

The intervention had sufficiently high levels of engagement to be used in a full randomized controlled trial to test its effectiveness in improving levels of PA among people with SCI. The knowledge we gained from this pilot study informed improvements that were made in the full randomized controlled trial.

## Introduction

People living with spinal cord injury (SCI) experience increased risk for obesity, excessive fat mass, abnormal lipid metabolism, and glucose intolerance than the general population [1-5]. At the same time, people with SCI have lower activity levels by as much as 45% to 66% than those without disability and still lower levels than groups with other disabilities, such as cerebral palsy, leg amputation, and chronic heart failure [6,7]. Accumulating evidence has demonstrated the positive effects of physical activity (PA) among people with SCI on their fitness, muscle strength, body composition, function, psychological well-being, and quality of life, all of which may mitigate the risk of developing chronic disease in the long term [8-11]. Evidence supports the need to increase PA levels among people with SCI, while a previous review has demonstrated the lack of effective options to increase PA among this population [12].

People with SCI face unique barriers to PA and exercise, including lack of reliable and accessible transportation, which also limits their ability to use community-based recreation centers [13,14]. The lack of recreation facilities with accessible equipment and knowledgeable staff who can assist people with mobility impairments reduces options for exercise [15]. Other barriers include lack of funds to purchase appropriate exercise equipment or gym memberships, plus low self-efficacy for exercise among people with disabilities [14,16-19]. The internet offers a way to overcome these barriers and meet the needs of people with SCI and other disabilities through instant access to information, on demand from the location of their choice, including at home, with the added benefit of connecting with others remotely. Although internet access is lower among those with lower income and racial/ethnic groups, recent US data show that 69.2% of people with traumatic SCI use a computer regularly, and of those, 99.8% have internet access [20]. Despite the lack of access among some groups, current data demonstrate that an internet-based intervention has the potential to reach many participants who would benefit from increasing their PA.

As we have previously detailed [21], internet- or phone-based methods to connect with people with SCI are not yet in widespread use; however, the few reports in existence before this study demonstrated efficacy in providing health education [22], developing self-management skills [23], and reducing depressive symptoms along with pain [24] among people with SCI. Although these studies offer evidence of efficacy that technology-mediated interventions can improve health, to our knowledge, none have focused on using regularly scheduled teleconferencing to overcome transportation barriers and increase PA while facilitating social support among peers. We identified one study from the literature that attempted to facilitate social support virtually through online message boards while investigating the effectiveness of using a web-based intervention to increase PA among adults with physical disability [25]. However, the participants’ use of the boards was almost nonexistent, and the overall effectiveness of the intervention was inconclusive [25]. Although the authors noted that the exact reason for low use of the message boards was unknown, some participants stated that they were uncomfortable sharing personal information with strangers on the web [25].

**Figure 1:**
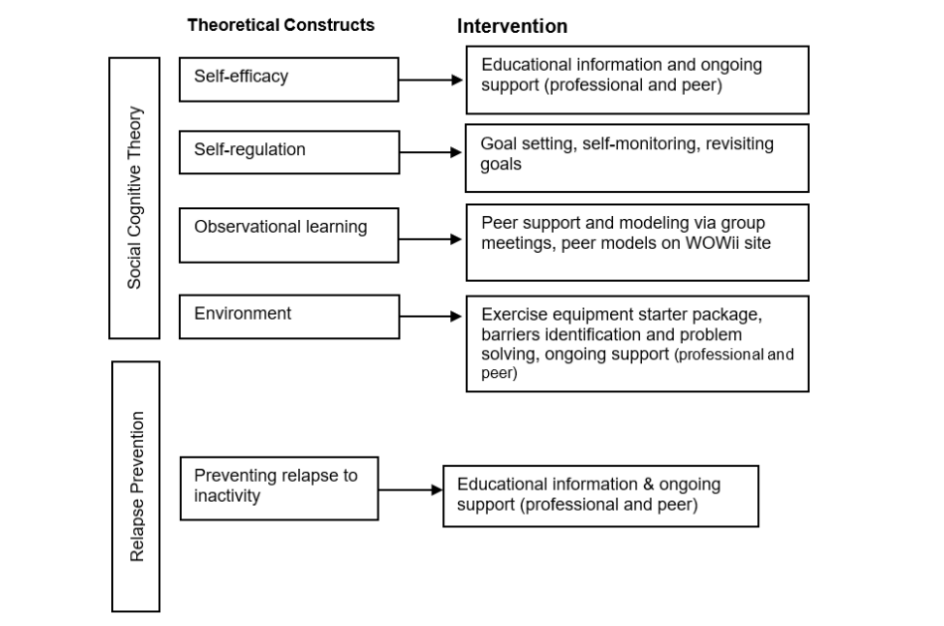
Theoretical constructs and key components of the Workout on Wheels internet intervention (WOW*ii*) program.

Our team created the Workout on Wheels internet intervention (WOW*ii*) program to meet the unique needs of people with SCI while delivering a theory-based health behavior intervention that facilitates videoconference meetings among groups of people with SCI interested in increasing PA [26]. This intervention was adapted from the Workout on Wheels (WOW) program, a telephone-based program that yielded significant increases in time spent performing aerobic exercise [27]. The WOW program was rooted in relapse prevention and social cognitive theories, both of which have been successfully implemented in interventions to increase PA [28]. Figure 1 depicts how each theory informs key components of the WOW*ii* interventions.

Although the WOW successfully increased PA, the one-on-one meetings conducted by telephone were very time intensive per participant and did not allow the participants to connect with one another, which was a highly requested feature [27]. The Workout on Wheels internet intervention (WOW*ii*) program translates the telephone-based WOW program into a web-based format delivered using web-based modules and virtual, group-based videoconference sessions. The advantages of WOW*ii* include leveraging technology to target groups of people with SCI to make resources available on demand while offering opportunities for participants to make personal connections throughout the intervention. This pilot study sought to characterize the feasibility of delivering the WOW*ii* program to 10 individuals with SCI based on the participants’ engagement in the program, defined as their attending weekly videoconference sessions, completing weekly online modules, and syncing their arm-based activity monitors weekly.

## Methods

### Recruitment

This study was conducted at Baylor Scott & White Institute for Rehabilitation in Dallas, Texas. Recruitment began in late 2016, and the study activities were concluded by May 2017. The Baylor Scott & White Research Institute human subjects committee approved the study activities (Institutional Review Board #016-091), and all participants provided informed consent prior to beginning study participation. Eligible participants were between 18 and 70 years of age and were not pregnant; lived with an SCI at the level of C5 or below for more than 6 months; used a wheelchair; spoke and read English; had a computer and internet access; and could independently navigate the study website. Former patients with SCI from our inpatient rehabilitation hospital who agreed to be contacted for future studies were invited to participate in the study via telephone call or email, and staff handed out fliers at the outpatient clinic and the monthly support group for people with SCI. Study staff screened potential participants for eligibility by telephone. For those deemed eligible to enroll, study staff obtained a medical clearance by fax to participate in an intervention targeting physical activity from each participant’s primary care provider. Participants in this pilot study were not eligible to continue to participate in the full randomized controlled trial (RCT) study, which is currently under review.

### Intervention and Data Collection

The WOW*ii* intervention comprises three key components: (1) the WOW*ii* exercise program provided via modules on the WOW*ii* website, (2) a starter package of exercise equipment, and (3) weekly virtual videoconference meetings led by the study staff to facilitate support for exercise. Study staff who led the videoconference meetings held master’s degrees in public health. They were trained to deliver the intervention and supervised by the principal investigator, an applied behavioral psychologist who has delivered exercise interventions to people with disabilities for over 20 years.

**Figure 2:**
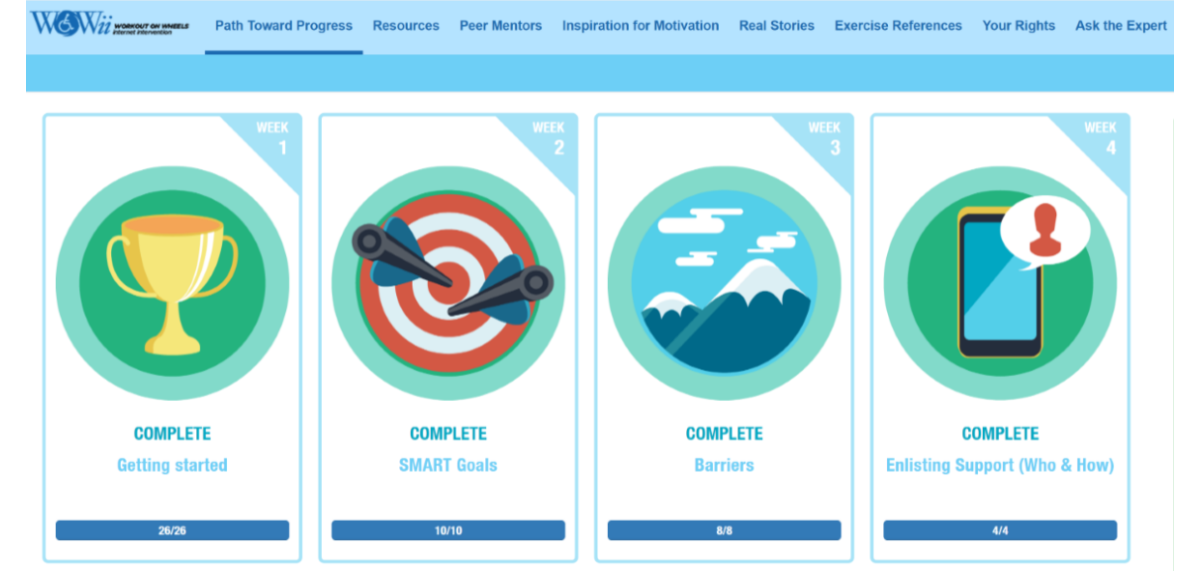
Workout on Wheels internet intervention (WOW*ii*) website and module topics covered during the pilot study.

For this pilot study, participants were given a username and password to access 4 weeks of the WOW*ii* website modules, which addressed getting started (eg, importance of exercise to health, benefits for people with disabilities, progression and safety, exercise basics), goal setting, identifying and addressing barriers to activity, and enlisting support (Figure 2). There was an activity at the end of each module that was designed to reinforce the material covered. Screenshots of the website and activities are available to view in Multimedia Appendices 1-4. The WOW*ii* website uses an application programming interface to collect data from each participant’s Polar account, allowing the study staff to download participant exercise data weekly in addition to their completed module activities.

The starter package of exercise equipment included resistance bands, an aerobic exercise DVD, and a pedal exerciser. Participants were also given Polar A300 wristwatch activity monitors [29] and Polar H7 heart rate monitors [29] (Polar Electro Oy) for recording the frequency and intensity of exercise bouts. These devices operate with proprietary Polar software, Polar Flow [30], which tracks and displays its data on the watch that can be downloaded through a compatible smartphone app or a web browser directly from the Polar Flow website.

Weekly videoconference meetings conducted over Skype were facilitated by a study coordinator and/or the principal investigator. The content of these discussions was designed to reinforce the information covered during each weekly module from the website. The format of each session provided time for the leader to briefly review the key points of the web-based module while facilitating conversation among the group regarding their experiences with each module topic. At the end of each session, leaders prompted the participants to complete the weekly module activity that reinforced the material discussed and asked participants to share their activity responses with the group. Session leaders recorded weekly attendance in a web-based spreadsheet.

### Analyses

Summary statistics were generated to describe the levels of individual and overall participant engagement as measured by percentage of sessions attended, percentage of weeks of exercise tracking via the Polar device, and percentage of website module activities completed. The data were summarized using SAS, version 9.4 (SAS Institute).

## Results

A convenience sample of 10 people were enrolled in the pilot trial and provided engagement data for analysis. The sample was mostly male (6/10, 60%) and White (9/10, 90%) with an average age of 38.8 years (SD 16.6), and the participants had lived with their SCI for an average of 4.2 years (SD 2.6) (Table 1). A slight majority (6/10, 60%) were not currently employed, and most reported education levels of technical school/some college or a high school diploma (6/10, 60%). Of the 10 participants, 7 (70%) reported cervical-level injuries, with equal numbers of power and manual wheelchair users.

**Table 1:** Participant demographics (N=10).

Variable		Value
Age (years), mean (SD)		38.8 (4.2)
Time with Injury (years), mean (SD)		16.6 (2.6)
**Sex, n (%)**		
	Male	6 (60)
	Female	4 (40)
**Race, n (%)**		
	White	9 (90)
	Other	1 (10)
**Ethnicity, n (%)**		
	Non-Hispanic	10 (100)
**Education level, n (%)**		
	Bachelor's degree or higher	4 (40)
	High school or below	3 (30)
	Technical school/some college	3 (30)
**Employment status, n (%)**		
	Employed full- or part-time	4 (40)
	Not currently working	6 (60)
**Income level (US $), n (%)**		
	0-39,000	6 (60)
	40,000-69,000	1 (10)
	>100,000	3 (30)
**Injury level, n (%)**		
	Cervical	7 (70)
	Thoracic	3 (30)
**Wheelchair type, n (%)**		
	Manual	5 (50)
	Power	5 (50)

Tables 2 and 3 demonstrate the level of overall engagement across the 10 participants. A total of 2 participants had low program engagement in the key components (2/12, 17%, and 5/12, 42%, respectively), with the other 8 demonstrating engagement rates of 75% to 100%. All participants attended at least 2 sessions; average videoconference session attendance ranged from 80% to 90% over the first 3 weeks and dropped to 70% in the fourth week. Most participants (9/10, 90%) completed the weekly web-based activities; however, only 70% (7/10) completed the final week’s activity. Of the total sample, 90% (9/10) synced their arm-based Polar activity monitors, with at least 60% (6/10) syncing the first 3 weeks; however, only 40% (4/10) synced the watch in the fourth week. The participants’ overall engagement as an average of each component across the 4 weeks was 75%. One participant only attended 2 virtual videoconference sessions and did not engage with the other components, and one participant completed 100% of the activities.

**Table 2:** Participant engagement in key intervention components (N=12) over the 4-week pilot.

Participant #	Activities	Week 1	Week 2	Week 3	Week 4	Overall engagement, n (%)
201	Attended session	✓	✓	✓	✓	11 (92)
	Completed activity	✓	✓	✓	✓	
	Tracked exercise		✓	✓	✓	
202	Attended session	✓	✓	✓	✓	11 (92)
	Completed activity	✓	✓	✓	✓	
	Tracked exercise	✓	✓	✓		
203	Attended session	✓		✓		2 (17)
	Completed activity					
	Tracked exercise					
204	Attended session	✓	✓	✓	✓	12 (100)
	Completed activity	✓	✓	✓	✓	
	Tracked exercise	✓	✓	✓	✓	
205	Attended session		✓	✓		5 (42)
	Completed activity	✓	✓	✓		
	Tracked exercise					
206	Attended session	✓	✓	✓	✓	11 (92)
	Completed activity	✓	✓	✓	✓	
	Tracked exercise	✓	✓	✓		
207	Attended session	✓	✓		✓	10 (83)
	Completed activity	✓	✓	✓	✓	
	Tracked exercise	✓	✓	✓		
208	Attended session	✓	✓	✓	✓	10 (83)
	Completed activity	✓	✓	✓	✓	
	Tracked exercise			✓	✓	
209	Attended session	✓	✓	✓		9 (75)
	Completed activity	✓	✓	✓		
	Tracked exercise	✓	✓	✓		
210	Attended session	✓	✓		✓	9 (75)
	Completed activity	✓	✓	✓	✓	
	Tracked exercise	✓	✓			

**Table 3:** Average engagement for the 3 components of the intervention.

Component	Average engagement, n (%)					
	Week 1	Week 2	Week 3	Week 4	Overall	
All components (total=30)	24 (80)	25 (83.3)	24 (80)	17 (56.7)	22.5 (75)	
Session attendance	9 (90)	9 (90)	8 (80)	7 (70)	33 (82.5)	
Online activity completion	9 (90)	9 (90)	9 (90)	7 (70)	34 (85)	
Exercising tracking rate	6 (60)	7 (70)	6 (60)	4 (40)	23 (57.5)	

## Discussion

### Principal Findings

Although individual participation levels varied from week to week, the participants demonstrated consistent engagement during the 4-week pilot, with an overall engagement rate among the sample of 75% for attending the weekly videoconference meetings, completing the web-based activities, and wearing and syncing the arm-based Polar activity monitor. The participants showed the highest rates of engagement for completing the web-based module activities (85%) and attending the videoconference sessions (83%). These activities offer participants the space to learn about behavioral skills critical to successful behavior change and to discuss with the group their plans and personal experiences implementing those plans. The rate of syncing the Polar activity monitors was lower at 56%, although 90% of participants synced the watch in at least 1 week. It was unclear whether participants had trouble syncing their watches or were not exercising each week.

This pilot study offers preliminary evidence that a web-based platform may be feasible for delivering a PA intervention program to meet the unique needs of people with SCI. Feedback from participants in this feasibility study was vital in implementing changes to the final website used during the RCT. For example, participants recommended incorporating a more robust peer mentoring component than we originally envisioned. The participants emphasized the importance of having individuals with SCI available to discuss their successes in increasing PA as a motivational piece for those starting the program. Additionally, participants advocated for adding cost-effective resources to the website, such as equipment and fitness programs, to best accommodate those whose disability may have limited access to disposable income. After conducting this initial feasibility study, the research team initiated testing the full 16-week theory- and evidence-based program in a randomized controlled trial with 168 total participants with SCI. That trial offered a more robust test of the effectiveness of the program in promoting increased moderate-intensity PA among a large SCI sample as well as the program’s effects on the participants’ self-reported self-efficacy and barriers to engaging in health promoting behaviors.

In contrast with the study by Kosma et al [25], which included a web-based message board that received only 6 postings over the course of the study, attendance at the virtual videoconference sessions was relatively high at 83%. During these weekly sessions, we observed that the participants were actively engaged in the conversation, and several reported that their favorite part of the program was connecting with other people with SCI. We believe that the high engagement we observed was due to participants being able to see one another and connect by videoconference, which mitigated any discomfort participants may have felt with sharing information on the web. Notably, a study published in 2019 that obtained qualitative feedback regarding a web-based portal aimed at increasing self-efficacy for exercise among a population with SCI reported that participants endorsed many of the features present in our program, such as self-regulation strategies, knowledge, and action planning [31].

Our observed attrition rate of 20% falls within an expected and acceptable range based on the previous WOW trial and research on weight loss and internet-based interventions. The WOW trial saw a 33% attrition rate [27], while a 2011 systematic review examining dropout among participants of intensive lifestyle weight loss interventions (61 total studies) found that the reported attrition rates among participants ranged from 9% to 90% depending on the length and type of the intervention [32]. Other systematic reviews examining participation in internet-based interventions for anxiety and depression found attrition rates of up to 83% [33,34].

### Limitations

A potential study limitation was the lack of racial diversity among the participants despite good diversity in terms of education and income. Additionally, initial problems with Polar watch data exports limited our ability to accurately measure exercise in minutes. However, this pilot study yielded valuable knowledge that was implemented in the full RCT. For example, due to confusion regarding the difference between PA and exercise, more information was added to the first website module. Changes were also made to bolster the participants’ interaction by having peer mentors with SCI join virtual sessions, content was conveyed by adding more informational videos, and other content was added about important disability-related legislation.

### Conclusions

The WOW*ii* program incorporated the following three novel components to increase PA among people with SCI: (1) group videoconferencing to leverage social support, (2) a web-based portal for information related to increasing PA, and (3) provision of exercise equipment to facilitate PA while mitigating barriers. Our pilot study showed promise regarding the ability of WOW*ii* to engage participants. The pilot study also led to improvements that were made before implementing the full RCT to study the effectiveness of the intervention. Given the prevalence of transportation issues and computer use among people with SCI, this intervention may be a valuable contribution to address the scarcity of lifestyle interventions which are accessible to this population.

## Appendix

Multimedia Appendix 1 Screenshot of Module 1 (Getting Started) activity.

Multimedia Appendix 2 Screenshot of Module 2 (SMART Goals) activity.

Multimedia Appendix 3 Screenshot of Module 3 (Barriers) activity.

Multimedia Appendix 4 Screenshot of Module 4 (Enlisting Support) activity.

## Abbreviations

PA: physical activity
RCT: randomized controlled trial
SCI: spinal cord injury
WOW: Workout on Wheels
WOW*ii*: Workout on Wheels internet intervention
