# Supplementary figures and images for: Feasibility of an Internet-Based Intervention to Promote Exercise for People With Spinal Cord Injury: Observational Pilot Study
Source: JMIR Rehabil Assist Technol. 2021 Jun 9;8(2):e24276. doi: 10.2196/24276 (PMC8235292; doi:10.2196/24276)

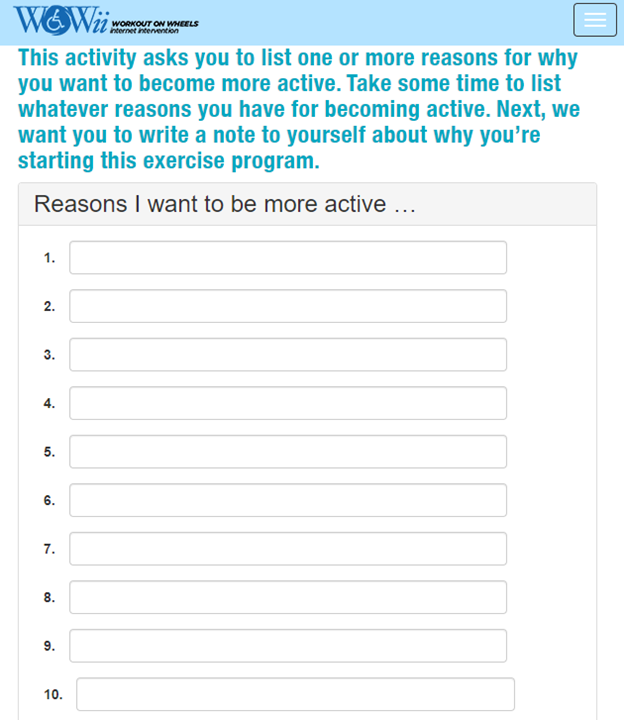

Supplement: Multimedia Appendix 1 [file rehab_v8i2e24276_app1.png]

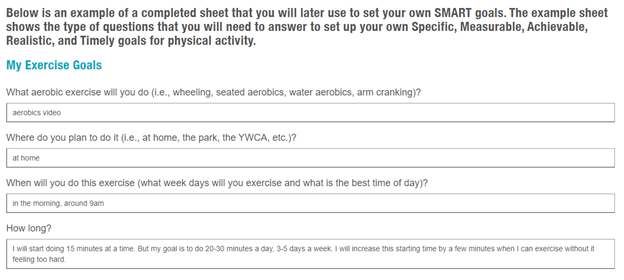

Supplement: Multimedia Appendix 2 [file rehab_v8i2e24276_app2.png]

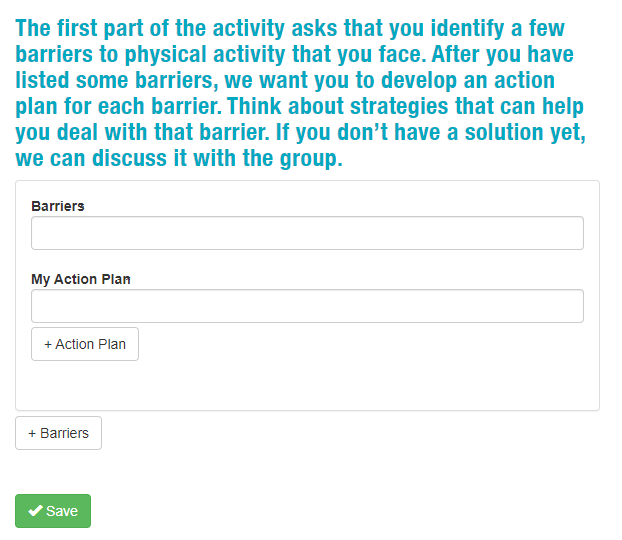

Supplement: Multimedia Appendix 3 [file rehab_v8i2e24276_app3.png]

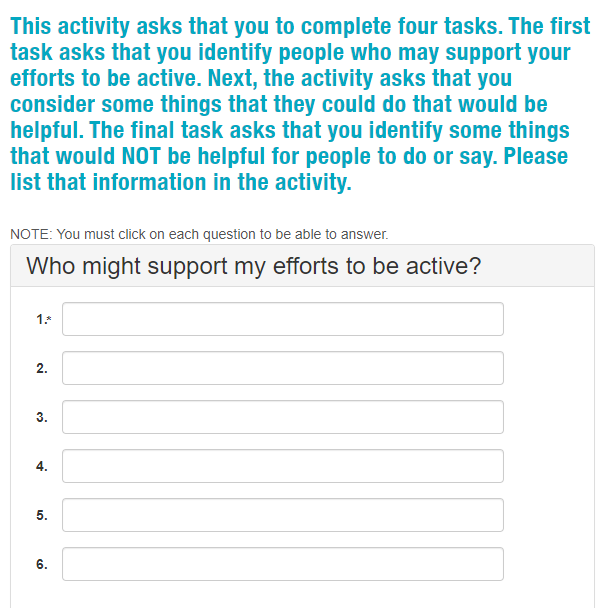

Supplement: Multimedia Appendix 4 [file rehab_v8i2e24276_app4.png]
